# Identification of a Human Protein-Derived HIV-1 Fusion Inhibitor Targeting the gp41 Fusion Core Structure

**DOI:** 10.1371/journal.pone.0066156

**Published:** 2013-05-31

**Authors:** Lijun Chao, Lu Lu, Hengwen Yang, Yun Zhu, Yuan Li, Qian Wang, Xiaowen Yu, Shibo Jiang, Ying-Hua Chen

**Affiliations:** 1 Laboratory of Immunology, School of Life Sciences, Tsinghua University, Beijing Key Laboratory for Protein Therapeutics, Protein Science Laboratory of the Ministry of Education, Beijing, P. R. China; 2 Key Laboratory of Medical Molecular Virology of Ministries of Education and Health, Shanghai Medical College and Institute of Medical Microbiology, Fudan University, Shanghai, P. R. China; 3 Laboratory of Viral Immunology, Lindsley F. Kimball Research Institute, New York Blood Center, New York, New York, United States of America; Fudan University, China

## Abstract

The HIV-1 envelope glycoprotein (Env) gp41 plays a crucial role in the viral fusion process. The peptides derived from the C-terminal heptad repeat (CHR) of gp41 are potent HIV fusion inhibitors. However, the activity of these anti-HIV-1 peptides *in vivo* may be attenuated by their induction of anti-gp41 antibodies. Thus, it is essential to identify antiviral peptides or proteins with low, or no, immunogenicity to humans. Here, we found that the C-terminal fragment (aa 462–521) of the human POB1 (the partner of RalBP1), designated C60, is an HIV-1 fusion inhibitor. It bound to N36, the peptide derived from the N-terminal heptad repeat (NHR) of gp41, and to the six-helix bundle (6-HB) formed by N36 and C34, a CHR-peptide, but it did not bind to C34. Unlike the CHR-peptides, C60 did not block gp41 6-HB formation. Rather, results suggest that C60 inhibits HIV-1 fusion by binding to the 6-HB, in particular, the residues in the gp41 NHR domain that are exposed on the surface of 6-HB. Since 6-HB plays a crucial role in the late stage of fusion between the viral envelope and endosomal membrane during the endocytic process of HIV-1, C60 may serve as a host restriction factor to suppress HIV-1 entry into CD4+ T lymphocytes. Taken together, it can be concluded from these results that C60 can be used as a lead for the development of anti-HIV-1 therapeutics or microbicides for the treatment and prevention of HIV-1 infection, as well as a molecular probe to study the fusogenic mechanism of HIV-1.

## Introduction

Acquired immune deficiency syndrome (AIDS) is caused by human immunodeficiency virus (HIV) and is one of the most important diseases threatening human health [1]. So far, more than 30 anti-HIV drugs have been licensed for treatment of HIV infection, including twelve reverse transcriptase inhibitors (RTIs), ten protease inhibitors (PIs), one integrase inhibitor, two entry inhibitors, and five combinatorial drugs [2]. T20 (brand name: Fuzeon; generic name: Enfuvirtide) is the only HIV entry inhibitor targeting the HIV-1 envelope glycoprotein (Env) transmembrane subunit gp41 for treatment of HIV/AIDS patients who fail to respond to the RTIs and PIs [3], [4]. Application of T20 *in vivo* has resulted in significant reduction of viral load [5], [6]. However, its clinical application is limited because the high (90 mg) drug dosage, which is injected subcutaneously twice daily, leads high cost to patients and serious local injection reactions. Several new peptides derived from the gp41 CHR with improved efficacy and half-life have been identified. However, administration of these peptides may lead to the production of antibodies against these peptides, which may attenuate their anti-HIV-1 activity [7]. Therefore, it is essential to develop anti-HIV-1 molecules with low, or no, immunogenicity to humans. One of the approaches is to identify human protein-derived antiviral agents.

It has been reported that several human proteins serve as host restriction factors to inhibit or block HIV-1 replication [8]. For example, the apolipoprotein B mRNA-editing catalytic polypeptides APOBEC3F and APOBEC3G are effective in inhibiting HIV-1 DNA integration [9]. Human and monkey tripartite motif-containing protein 5 alpha (TRIM5alpha) could restrict HIV-1 infection in humans and Old World monkeys, respectively [10]. Tetherin is able to prevent release of the HIV-1 particles from the surface of producer cells [11], [12]. The HECT domain and RCC1-like domain-containing protein 5 (HERC5) effectively restrict HIV-1 assembly at the late stage of the HIV-1 life cycle [13]. Although all the above human restriction factors can be developed as anti-HIV-1 therapeutics, none of them is effective in suppressing HIV-1 fusion and entry at the early stages of the HIV-1 life cycle.

**Figure 1:**
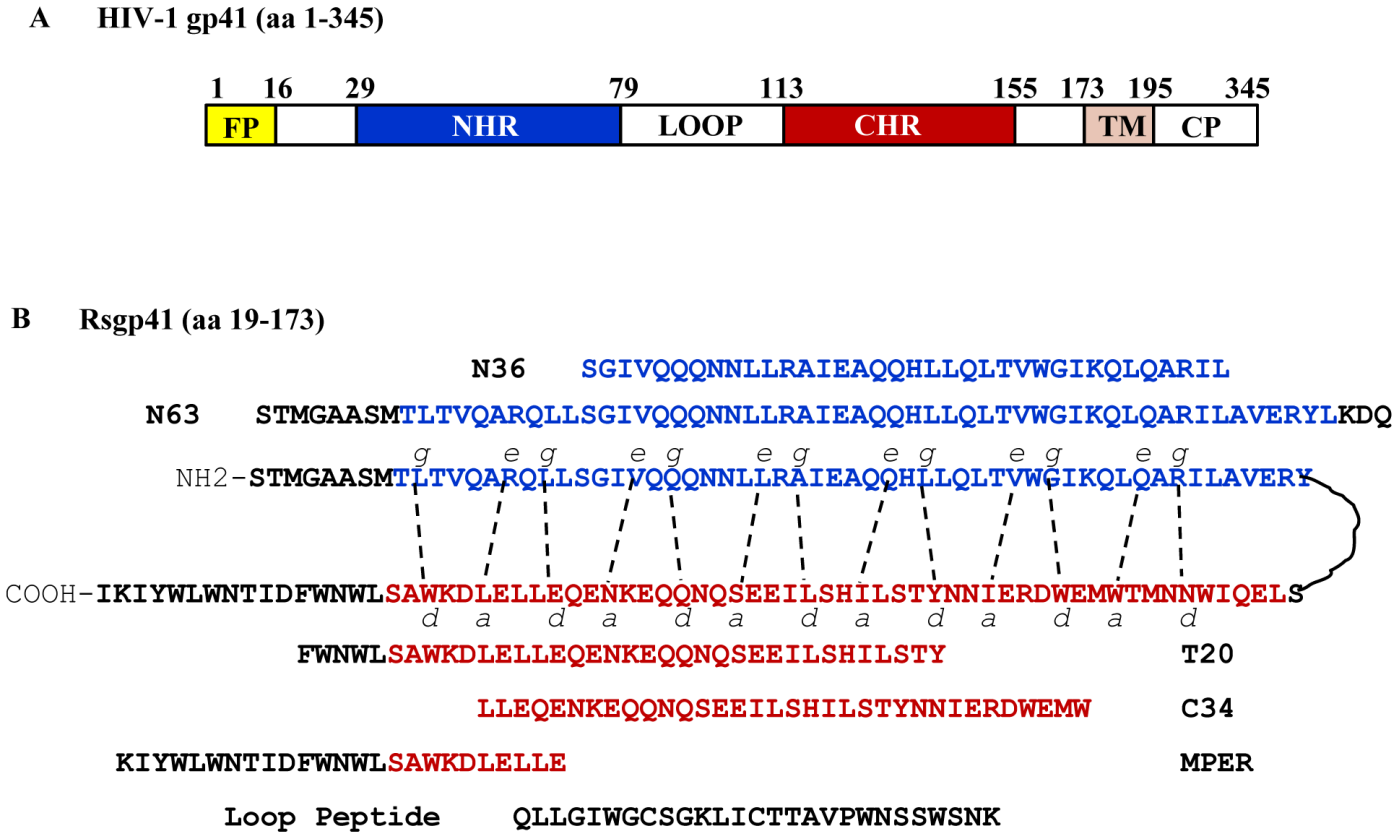
Schematic representation of HIV-1 gp41 and rsgp41. (**A**) Functional domains of the HIV-1 gp41. FP, fusion peptide; NHR, N-terminal heptad repeat; CHR, C-terminal heptad repeat; TM, transmembrane domain; CP, cytoplasmic domain. (**B**) Schematic representation of rsgp41. The dashed lines between the gp41 NHR and CHR domain indicate the interactions between the residues at the *e* and *g* positions in NHR and the *a* and *d* positions in CHR. The sequences of N36, C34, T20, MPER, N63, MPER and Loop peptide are shown.

HIV-1 entry is initiated by binding of the Env surface subunit gp120 with CD4 and a co-receptor, CCR5 or CXCR4, on the target cells [14], [15], triggering the conformation changes of gp41 from native state to pre-hairpin fusion intermediate, fusogenic and post-fusion states, sequentially. During the fusogenic state, some researchers believe that the interaction between the gp41 N- and C-terminal heptad repeat (NHR and CHR, respectively) domains (Fig. 1A) results in the formation of a six-helix bundle (6-HB) core structure on the target cell surface to bring the viral and target cell membranes into proximity for fusion [16]–[19]. The peptides derived from the gp41 CHR domain, such as C34 and T20, can bind with the viral gp41 NHR domain (Fig. 1B) to block viral gp41 6-HB core formation, thus inhibiting gp41-mediated membrane fusion [16]–[19]. However, Melikyan and colleagues have demonstrated that the gp41 6-HB core is not a dead-end structure, but may still play a role in the late stage of membrane fusion in the endocytic process of HIV-1, particularly since 6-HB actually forms immediately after fusion pore formation in the endosomal membrane after the HIV-1 particle has been rapidly endocytosed and internalized [20], [21]. These findings suggest that 6-HB can still serve as a target for HIV-1 fusion inhibitors.

Using the recombinant soluble gp41 ectodomain (rsgp41, Fig. 1B) as a bait to screen a human bone marrow cDNA library by the yeast-two-hybrid system, we identified an HIV-1 gp41-binding protein, human POB1 (the partner of RalBP1). Further dissecting the binding domains in both gp41 and POB1, we found that the fragment containing the C-terminal 60 amino acids of POB1 (aa462–521), designated C60, was responsible for the binding of POB1 to rsgp41 through the 6-HB core formed by the gp41 NHR and CHR domains. The peptide C60 could significantly inhibit HIV-1 Env-mediated cell-cell fusion and HIV-1 replication. Unlike the peptides derived from the gp41 CHR domain, C60 could not block the gp41 6-HB formation, but rather bound with 6-HB to halt further fusion. These results suggest that C60 inhibits HIV-1 entry into the host cell by targeting the late stage of the HIV-1 fusion, indicating its potential use as a lead for the development of a novel protein-based HIV fusion/entry inhibitor for treatment and prevention of HIV-1 infection. It can also be used as molecular probes for studying the role of 6-HB in the HIV-1 fusion process and the viral fusogenic mechanism.

## Materials and Methods

### Peptides, cells and viruses

The peptides T20, C34, MPER and Loop peptide (see their sequences in Fig. 1B) were all synthesized by SBS Genetech, Beijing, China, with purity >90%. 3T3 cells stably transduced with murine leukemia virus MX-CD4 and MX-CXCR4 vectors (3T3.T4.CXCR4) and 293T cells were cultured in DMEM medium complemented with 10% FBS, 100 IU/ml penicillin and 100 IU/ml streptomycin (Invitrogen, USA). CHO cells stably transfected with the HIV-1 HXB2 Env-expressing vector pEE14 (CHO-Env), or control pEE14 vector (CHO-C), were cultured in glutamine-deficient minimal essential medium (GMEM-S) containing 400 µM Methionine sulfoximine (Sigma, USA). MT-2 and TZM-b1 cells, HIV-1 IIIB and Bal strains, as well as the plasmids pHEF-VSVG and pNL4-3.luc.RE, were obtained from the NIH AIDS Research and Reference Reagent Program. The vesicular stomatitis virus glycoprotein (VSV-G) and influenza A virus hemagglutinin (IAV HA) pseudovirus were produced by cotransfecting 293T cells with pNL4-3.luc.RE and pHEF-VSVG or the plasmid encoding HA of IAV H5N1, respectively, using Lipofectamine™ 2000 (Invitrogen) as previously described [22], [23].

### Yeast-two-hybrid system

The BD Matchmaker yeast two-hybrid system (BD Bioscience Clontech, USA) was employed in this study, and the BD Matchmarker™ screen process followed instructions in the user manual, as described previously [24]. The rsgp41 (aa539–684) of HIV-1 HXB2 was cloned into the pGBKT7, which encodes Gal4 DNA-binding domain (BD), as the bait plasmid. The yeast strain AH109 was transformed with the bait plasmid and mated with yeast Y187 containing the human bone marrow cDNA library. The mated yeasts were screened on dropout media deficient in Try, Leu and His (-Try/-Leu/-His). Primary His+ colonies were rescreened on -Try/-Leu/-His/-Ade/X-Gal plates to confirm protein interaction. The library plasmids from the blue colonies were recovered and retested to eliminate false-positives. To locate the POB1/rsgp41 binding site, POB1 (400–521), POB1 (462–521) (also called C60) and POB1 (333–461) were amplified by PCR from the library plasmid pGADT7-Rec-POB1 (333–521) and cloned into the pGADT7 vector. These plasmids were transformed into pGBKT7-rsgp41, or control plasmid pGBKT7-T, containing yeast AH109 and grown on -Try/-Leu/-His/-Ade/X-Gal plates to test protein interaction.

### Recombinant protein expression and purification

PGEX-6p-1 vector, Glutathione Sepharose 4 Fast Flow Beads, and PreScission Protease (Amersham Pharmacia Biotech Inc., USA) were used to express and purify recombinant proteins with GST, tagged or not, as described previously [25]. For POB1 (333–461), POB1 (400–521), POB1 (462–521) and NC (N36-L8-C34), the DNA fragments were cloned into the vector. For the expression of 3NC, the NC DNA sequence was triplicated by the *Bam*HI and *Bgl*II fusion sites, as described previously [26]. The concentrations of purified proteins were calculated by analysis of Coomassie Brilliant Blue R-250-dyed SDS-PAGE with Band Leader software.

### Circular dichroism (CD) analysis

Spectra were obtained on a Jasco J-715 instrument with a 0.1 nm step size as described previously [27]. Samples (40–50 µM) were diluted in 10 mM Tris buffer (pH 7.4, containing 0.15 M NaCl and 3.4 mM EDTA) and placed in a 0.1 cm path-length CD cell. The spectra were averaged from four scans, blank corrected, smoothed and expressed as the mean residue ellipticity [θ] (MRE).

### Enzyme-linked immunosorbent assay (ELISA)

To determine C60 (POB1 (462–521)) and POB1 (333–461) binding activities to the rsgp41 domains, the ELISA assay was performed as described previously [28]. Briefly, wells of microtiter plates were coated overnight with 50 µl C60 or POB1 (333–461) (100 µg/ml) in 0.1 M NaHCO_3_ buffer (pH 9.6). The wells were then blocked with phosphate buffer solution (PBS, pH 7.4) containing 0.25% gelatin. After three washes with PBS containing 0.1% Tween-20 (PBS-T), rsgp41, N36(L8)C34, loop peptide, or MPER diluted in PBS were added separately, followed by incubation at room temperature for 1 h. After extensive washes, the amount of bound peptides was detected by addition of their rabbit antibody, peroxidase-conjugated anti-rabbit antibody and substrate, o-phenylendiamin (OPD), sequentially. The absorbance at 450 nm (A450) was recorded.

### Surface Plasmon Resonance (SPR) assay

The kinetics of the binding affinity between C60 and gp41 core was determined by SPR using the BIACORE X system (BIACORE, Sweden), following the biomolecular interaction analysis technology manual, as described previously [24]. Briefly, the CM5 sensor chip was immobilized with 3NC (100 µg/ml) by amine coupling, and the unreacted sites were blocked with ethanolamine. The association reaction was initiated by injecting 40 µl C60 or POB1 (333–461) at a flow rate of 20 µl/min. The dissociation reaction was performed by washing with running buffer (10 mM HEPES, pH 7.4, containing 0.15 M NaCl, 3.4 mM EDTA and 0.005% v/v Surfactant P20). At the end of the cycle, the sensor chip surface was regenerated with 10 mM HCl for 30 s.

### The interaction between C60 and HIV-1 gp41 expressed on CHO-Env cells

CHO-Env cells and the control CHO cells were prestimulated with 6.5 mM sodium butyrate for 20 hours and washed with PBS buffer (containing 1% FBS) three times. They were incubated with different concentrations of C60-FITC (0 µg/ml, 10 µg/ml, 50 µg/ml and 100 µg/ml) and recombinant soluble CD4 (sCD4, 50 µg/ml) at 4°C for 1 h. Conformation-specific monoclonal antibody (MAb) NC-1 [29] was used as a control. The fluorescence intensity was examined by a FACSCalibur flow cytometer (Becton Dickinson) after extensive washes.

### Fluorescence native polyacrylamide gel electrophoresis (FN-PAGE)

To determine whether C60 could interrupt gp41 6-HB formation, FN-PAGE was performed as described previously [30]. Briefly, C60, or the small-molecule fusion inhibitor ADS-J1 [31], [32] (100 µM), was mixed with N36 in equal volumes at 37°C for 30 min. Then C34-fluorescein isothiocyanate (FITC) was added into the mixture for another 30 min. After dilution with Tris-glycine native sample buffer (Invitrogen, Carlsbad, CA), the samples (20 µl) were loaded onto Tris-glycine gels (18%; Invitrogen, Carlsbad, CA), and the gels were run at a constant voltage of 120 V for 1 h at room temperature. After electrophoresis, the gels were observed and imaged by a FluorChem 8800 imaging system (Alpha Innotech Corp., San Leandro, CA) using a transillumination UV light source with an excitation wavelength of 302 nm and a fluorescence filter with an emission wavelength of 520 nm. The same gels were then stained with Coomassie Blue and imaged with the FluorChem 8800 imaging system using a visible light source.

### Detection of inhibition of 6-HB formation by ELISA

ELISA was used to analyze the inhibitory effect of C60 on the 6-HB formation as described previously [33]. Wells of microtiter plates were precoated with 100 µl mAb NC-1 (8 µg/ml) at 4°C overnight and blocked with 2% nonfat milk in PBS. C34 or C60 peptide in series concentrations and N36 or N63 (0.5 µM) were mixed at 37°C for 30 min. Then the mixture was added into the wells of microtiter plates with biotinylated C34 (C34-biotin, 0.5 µM) and incubated for 30 min at room temperature. Then horseradish peroxidase labeled with streptavidin (Zymed Laboratories, S. San Francisco, CA) was added into the wells. After extensive washing followed by adding the substrate TMB (3,3′,5,5′-tetramethylbenzidine; Sigma), absorbance at 450 nm (A_450_) was measured by an ELISA reader (Ultra 384; Tecan, Research Triangle Park, NC). The percent inhibition of 6-HB formation and the IC_50_s were calculated [34].

### Inhibition of HIV-1 Env-mediated syncytium formation by C60

5×10^4^ 3T3.T4.CXCR4 cells were incubated in wells of 48-well plates overnight. 3×10^4^ CHO-Env cells prestimulated with 6.5 mM sodium butyrate for 20 h were then added, with or without an inhibitor, at graded concentrations. The syncytia which were more than four times larger than single cells were counted under a microscope after being cultured for 24 h. The equation used to calculate the inhibition of syncytium formation was as follows: % inhibition = [1– (number of syncytia in a well containing an inhibitor)/(number of syncytia in a well containing no inhibitor)] ×100%. GST protein was used as a negative control, and the small-molecule fusion inhibitor ADS-J1 [31], [32] was used as a positive control. The concentration for 50% inhibition (IC_50_) was calculated using the CalcuSyn software [35].

### Inhibition of HIV-1 infection by C60

Inhibitory activities of C60 on infection by HIV-1 X4 strain IIIB were determined as described previously [36]. HIV-1 IIIB infected 200 µl MT-2 cells (1×10^4^/ml) at 100 TCID_50_ (50% tissue culture infective doses) with C60 at graded concentrations. After being cultured overnight, the medium was replaced with fresh medium. 100 µl culture supernatants were collected from each well on the fourth day and were mixed with equal volumes of 5% Triton X-100. The p24 antigen was detected by ELISA. Inhibitory activities of C60 on infection by HIV-1 R5 strain Bal and the VSV-G pseudovirus were determined as described previously [37]. HIV-1 Bal or VSV-G pseudovirus infected µl TZM-b1 cells (1×10^5^/ml) at 100 TCID_50_ with C60 at graded concentrations. 293T cells were used to determine the infectivity of the IAV HA pseudovirus. The luciferase activity of the lysed cells was analyzed using a luciferase kit (Promega, Madison, WI) and a luminometer (Ultra 386; Tecan, Durham, NC) on the fourth day. To determine whether the inhibition of HIV-1 infection by C60 is due to its influence on a cellular protein that regulates the endocytosis process, a “wash-out” assay was performed. TZM-bl cells (1×10^5^/ml) were cultured in wells of a 96-well plate overnight and incubated in the absence (PBS-treated) or presence of 100 µM of C60 (C60-treated) or 80 µM of MiTMAB (MiTMAB-treated) (MiTMAB is a dynamin inhibitor that can suppress the clathrin-mediated endocytosis) for 90 min at 37 °C. After incubation, the cells were washed with PBS and cultured with fresh medium containing HIV-1 Bal (100 TCID_50_) for 3 more days. The luciferase activity was determined as described above. The percent inhibition of luciferase activity and the IC_50_s were calculated using the CalcuSyn software [35].

### Analysis of cytotoxicity

The cytotoxicity of C60 on MT-2 and TZM-bl cells was measured by the Cell Counting Kit 8 (Dojindo). The cells were cultured with C60 at graded concentrations for 4 days in wells of 96-well plates, and washed twice with PBS. Then, 10 µl of WST-8 was added to each well. After incubation for 2 h, the absorbance at 450 nm was measured by an ELISA reader and the % cytotoxicity was calculated.

## Results

### Identification of the C-terminal region (C60, aa 462–521) of the human POB1 protein as the HIV-1 gp41 binding site

To identify a human protein that can physically interact with the ectodomain of gp41, we conducted a yeast two-hybrid screening of a human bone marrow cDNA library. From 5×10^6^ screened clones, a blue clone that grew on the -Try/-Leu/-His/-Ade/X-Gal plate was identified. The corresponding plasmid, which contained a 700 bp cDNA identical to the 3′ region of POB1 mRNA on the basis of nucleotide blasting on NCBI, was then recovered. This cDNA insert encodes a protein sequence corresponding to the C-terminal region (aa 333–521) of the POB1 protein.

**Figure 2:**
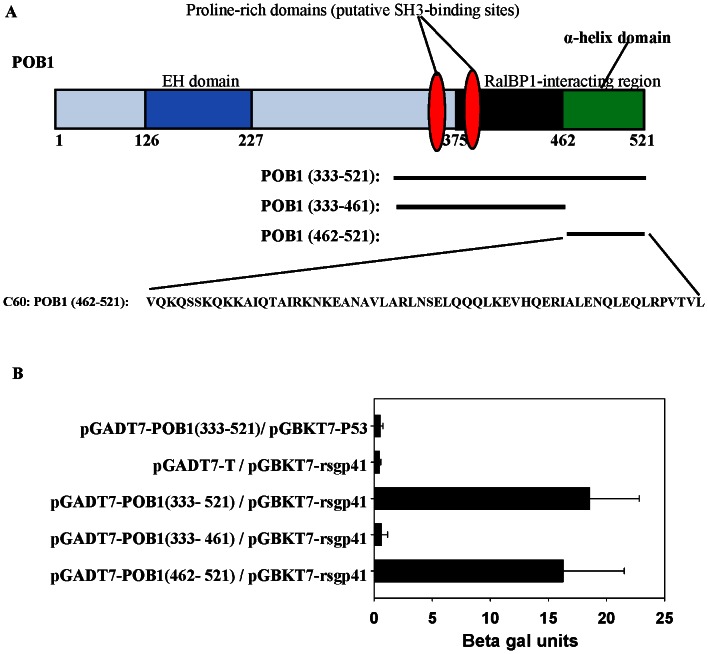
Identification of the C-terminal region (C60, aa462–521) of POB1 as a gp41 binding site. (**A**) POB1 (333–521) represents the coded fragment from the yeast two-hybrid. POB1 (462–521) (C60) corresponds to the predicted coiled-coil. POB1 (333–461) is the N-terminal fragment of POB1 (333–521) after C60 is removed. (**B**) The specific interaction between rsgp41 and C60 or POB1 (333–461) was determined by yeast two-hybrid assay.

According to previous report, this POB1 (333–521) region covers the RalBP1 binding domain (aa 375–521), which contains two putative SH3 binding sites and a predicted coiled-coil domain (Fig. 2A) [38]. To further identify the fragment in the POB1 (333–521) region that mediates binding to gp41, plasmids expressing POB1 (333–521), POB1 (333–461), and POB1 (462–521) that contain the predicted coiled-coil domain were constructed and tested for interaction with rsgp41 in the yeast two-hybrid system. Plasmids pGADT7-T and pGBKT7-P53 were used as control. Only yeast cells bearing the pGADT7-POB1 (333-521) or pGADT7-POB1 (462–521) plasmids that were cotransformed with pGBKT7-rsgp41 show His+/Ade+/Mel1+ phenotype, whereas the other yeast cells did not (Fig. 2B). These results indicate that the C-terminal fragment of POB1 (aa 462–521), designated C60, mediates the binding of POB1 to rsgp41.

### Inhibition of HIV-1 Env-mediated cell-cell fusion and HIV-1 infection by C60

**Figure 3:**
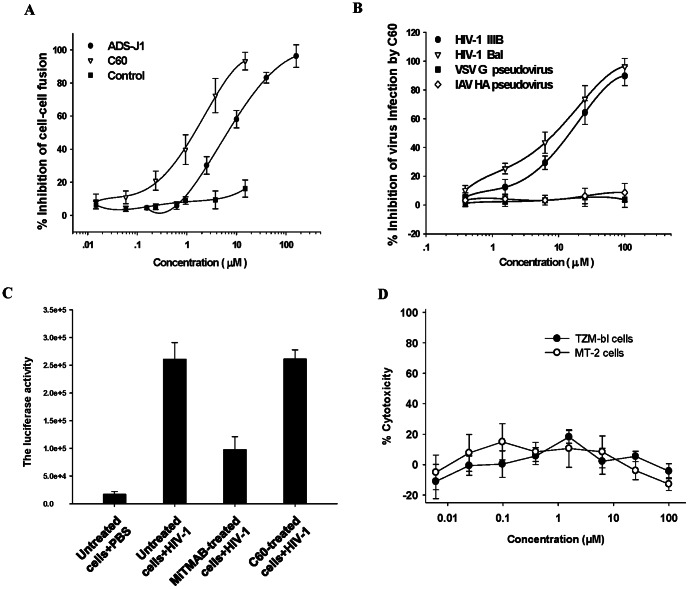
Inhibition activity of C60 on syncytium formation and HIV-1 infection. (**A**) Inhibition of C60 on HIV-1 Env-mediated syncytium formation. GST was used as the negative control, and ADS-J1 was used as the positive control. (**B**) Antiviral activity of C60 on the infection by HIV-1 IIIB (X4-tropic) and Bal (R5- tropic). The VSV-G and IAV HA pseudoviruses were used as control. (**C**) Susceptibility of TZM-bl cells pretreated with C60 and MiTMAB (a inhibitor of clathrin-mediated endocytosis). TZM-bl cells were pre-treated with PBS, C60 (100 µM) or MiTMAB (80 µM) and washed three times to remove the unbound inhibitors before addition of HIV-1 Bal for infection. The untreated cells incubated with PBS were included as control. The experiment was performed in triplicate and the data are presented in mean±SD. (**D**) Cytotoxicity of C60 on MT-2 and TZM-bl cells. Cells were incubated in absence or presence of C60 at graded concentrations for four days, followed by measurement of the cell viability using the Cell Counting Kit 8. The experiment was performed in triplicate and the data are presented in mean±SD.

Next, we expressed the polypeptide C60 and tested its inhibitory activity on HIV-1 Env-mediated cell-cell fusion using 3T3.T4.CXCR4 cells that express CD4 and CXCR4, as the target cells, and CHO-Env cells that express the HIV-1 Env, as the effector cells. We found that C60 is highly effective in inhibiting the HIV-1 Env-mediated cell-cell fusion in a dose-dependent manner. The IC_50_ was 1.09±0.39 µM (Fig. 3A), which is lower than that of ADS-J1, the positive control HIV-1 entry inhibitor.

Subsequently, we assessed the antiviral activity of C60 against infection by two laboratory-adapted HIV-1 strains, IIIB (X4-tropic) and Bal (R5-tropic). As shown in Fig. 3B, C60 could effectively inhibit infection by both HIV-1 IIIB and Bal with IC_50_ values of 15.28±4.56 µM and 9.21±3.25 µM, respectively, while it showed no inhibition of infection by either VSV-G or IAV HA pseudoviruses, suggesting that C60 can specifically interact with the HIV-1 Env and inhibit gp41-mediated membrane fusion and, hence, HIV-1 infection.

To determine whether the inhibition of HIV-1 infection by C60 is due to its influence on a cellular protein that regulates the endocytosis process, a “wash-out” assay was performed. As shown in Fig. 3C, TZM-bl cells pre-treated with C60 are still susceptible to HIV-1 Bal infection, while the cells pretreated with MiTMAB (a inhibitor of clathrin-mediated endocytosis) became resistant to the viral infection, suggesting that unlike MiTMAB, C60 does not interact with a cellular protein (e.g., clathrin or dynamin) to interfere with the cellular protein-regulated endocytosis process.

Subsequently, we tested the potential cytotoxicity of C60. As shown in Fig. 3D, C60 exhibited no significant cytotoxicity to MT-2 and TZM-bl cells at the concentration as high as 100 µM, suggesting that the HIV-1 fusion-inhibitory activity of C60 is not due to its cytotoxicity.

### Binding of C60 with the HIV-1 gp41 6-HB structure

**Figure 4:**
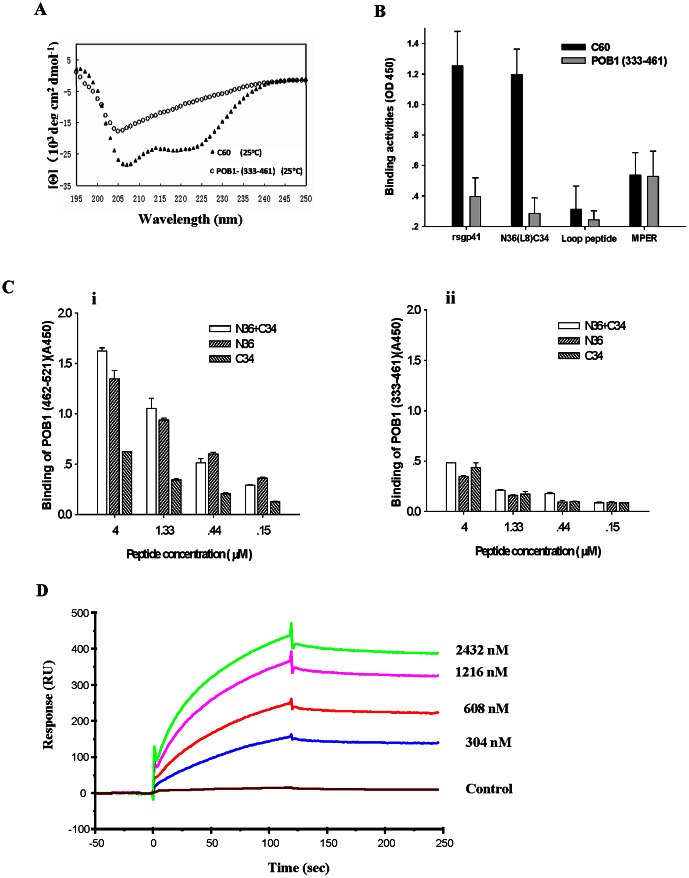
Binding activity of C60 and POB1 (333-461) with rsgp41. (**A**) CD spectra of C60, which represents the coiled-coil conformation, while POB1 (333–461) does not. (**B**) ELISA analysis to determine the binding site of regp41 interacting with C60. POB1 (333–461) was used as control. (**C**) ELISA analysis of C60 interaction with gp41 fusion core. Wells of microtiter plates were coated with purified C60 (i) or POB1 (333–461) (ii). Reaction with serial concentrations of N36 or C34 peptides, or the complex containing half the quantity of each peptide. (**D**) Binding affinity of C60 to gp41 fusion core analyzed by SPR. Series concentrations of purified C60 were allowed to flow pass the control channel (without 3NC coating) and the immobilized channel, sequentially. The *K_d_* value was calculated by BIAevaluation 3.1 software. POB1 (333–461) control shows no interaction with gp41 fusion core.

In order to determine the secondary conformation of C60, recombinant C60 protein was analyzed by CD spectra. The CD spectrum of C60 exhibited negative troughs at 222 and 208 nm (Fig. 4A), confirming its coiled-coil confirmation, which is consistent with the prediction from bioinformatics analysis [38].

To determine which domain of rsgp41 is responsible for binding to C60, we performed an ELISA assay using rsgp41, N36(L8)C34, and peptides derived from the gp41 loop region and MPER. The results showed that only rsgp41and N36(L8)C34 could strongly bind to C60 (Fig. 4B). Importantly, since N36(L8)C34 has been recognized as a model of the gp41 6-HB core [39], the above results implied that the C60-binding site in rsgp41 is the gp41 6-HB fusion core. Using ELISA, we then further tested the binding activity of C60 with the gp41 N-peptide N36, C-peptide C34 and N36/C34 complex at graded concentrations. The results also proved that C60 bound strongly to the gp41 6-HB formed by N36 and C34, as well as the N36 peptide (Fig. 4C), suggesting that the C60 helix may bind to some sites in gp41 NHR that are exposed on the surface of the gp41 6-HB.

Real-time surface plasmon resonance (SPR) was then employed to analyze the kinetic characteristics of the affinities between C60 and gp41. We generated a recombinant protein 3NC, which contains a triplicate N36-GGSGG-C34 where GSSGG serves as a linker between N36 and C34 peptides [40] to obtain a more stable gp41 6-HB core. The purified 3NC protein was immobilized on the surface of a carboxymethylated dextran sensor chip (CM5). Purified C60 with serial concentrations were allowed to flow through the control channel without 3NC coating and the 3NC-immobilized channel, sequentially, and the interaction was recorded as the different respondent spectra on the sensorgram. The data were simulated with the BIAevaluation 3.1, and the resulting equilibrium constant (*K_d_*) of the interaction between 3NC and C60 was calculated to be at 1.55e^−8^ M (Fig. 4D). This result confirmed the strong interaction between C60 and gp41 6-HB.

### Interaction of C60 with the CD4-induced gp41 core on the cell surface

**Figure 5:**
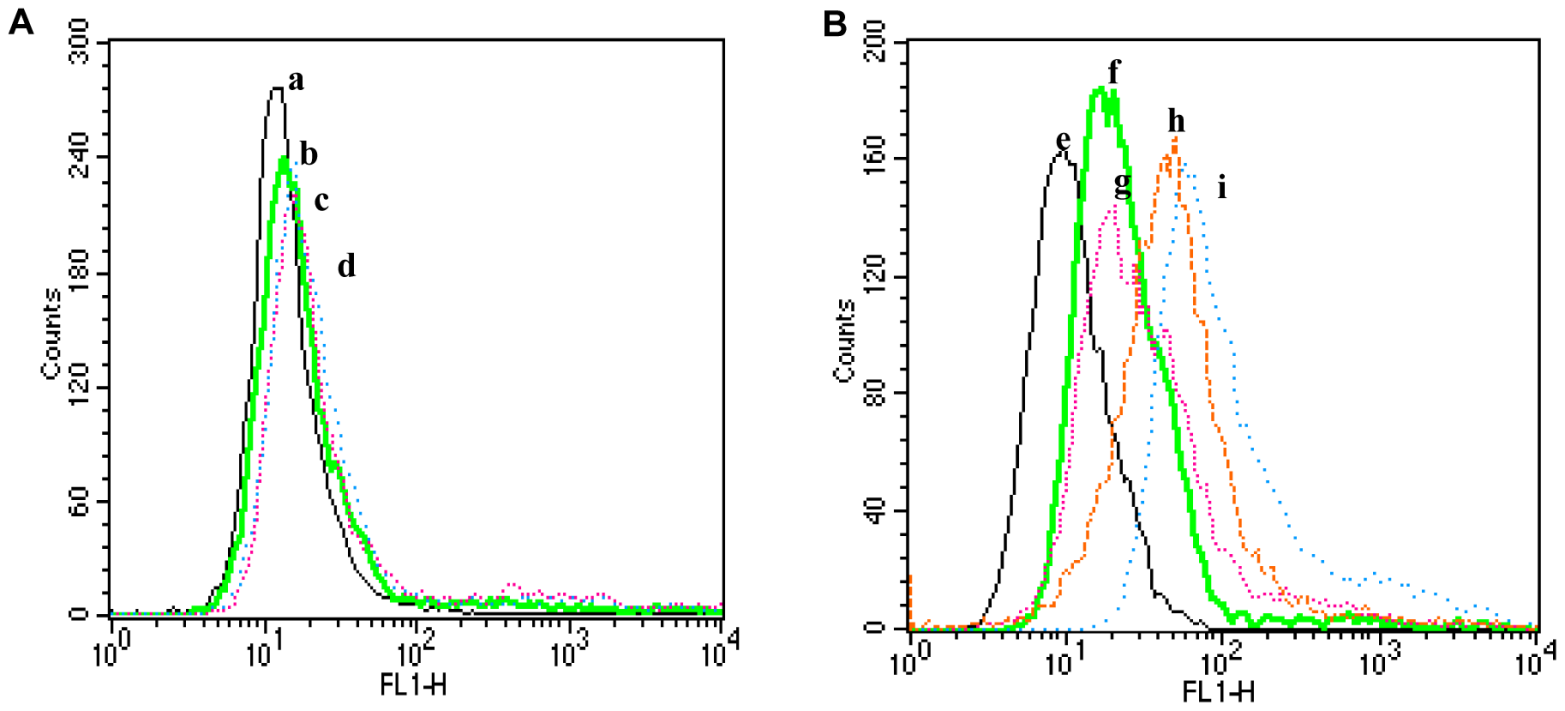
Binding of C60-FITC to HIV-1 gp41 expressed on CHO-Env cells. (**A**) CHO-C cells without gp160 were reacted with different concentrations of C60-FITC (a, 0 µg/ml; b, 10 µg/ml; c, 50 µg/ml) in the presence of sCD4 (50 µg/ml) and d, NC-1 mAb (50 µg/ml). (**B**) CHO-Env cells with gp160 were reacted with different concentrations of C60-FITC (e, 0 µg/ml; g, 10 µg/ml; h, 50 µg/ml; i, 100 µg/ml) in the presence of sCD4 (50 µg/ml) and f, NC-1 mAb (50 µg/ml).

Having confirmed the interaction between C60 and the gp41 6-HB, a flow cytometry assay was then used to determine the specific interaction between C60 and HIV-1 gp41 expressed on CHO-Env cells. Recombinant soluble CD4 (sCD4) was used to dissociate gp120 from gp41 and expose gp41 6-HB on the cell surface [41]. In the control CHO cells, the fluorescence intensity presents no difference between samples with different concentrations of C60-FITC. However, in the sCD4-treated CHO-Env cells, samples did present florescence intensity corresponding to the concentrations of C60-FITC, indicating that C60 is able to interact with gp41 6-HB core in a dose-dependent manner (Fig. 5).

### Inability of C60 to disrupt the gp41 6-HB formation

**Figure 6:**
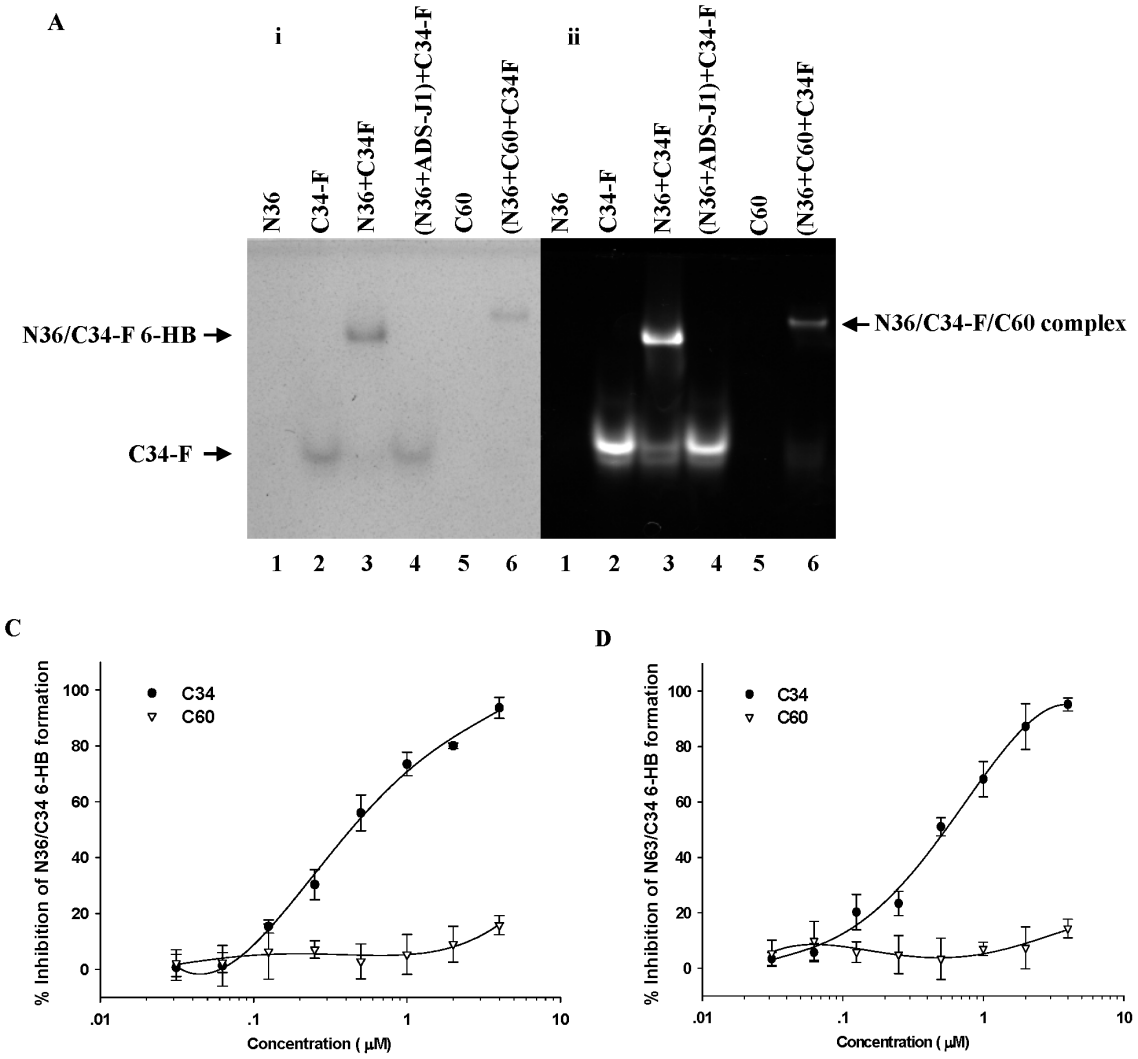
Inhibition activity of C60 on 6-HB formation. (**A**) FN-PAGE gel was stained with Coomassie Blue (i) or was visualized using a transillumination UV light source (ii). Line1, N36; Line 2, C34-FITC; Line 3, N36/C34-FITC; Line 4, N36/C34-FITC/ADS-J1; Line 5, C60; Line 6, N36/C34-FITC/C60. (**B**) ELISA was used to determine the inhibition effect of C60 on gp41 6-HB formation by N36 and C34-biotin. C34 was used as control. (**C**) ELISA was used to determine the inhibition effect of C60 on gp41 6-HB formation by N63 and C34-biotin. C34 was used as control.

We carried out an FN-PAGE to test whether C60 could block the formation of 6-HB by the peptides N36 and C34-FITC. ADS-J1, a small-molecule HIV-1 fusion inhibitor able to disrupt the 6-HB formation [31], [32], was used as a positive control. The gel was visualized by staining with Coomassie Blue (Fig. 6A1) or UV light (Fig. 6A2). As expected, N36 peptide alone (lane 1) showed no band because N36 carries net positive charges, thus migrating up and off the gel, while C34-FITC exhibited a band at the lower position in the gel (lane 2) [31], [32]. The N36/C34-FITC mixture displayed a band at the upper position in the gel (lane 3), corresponding to the band of 6-HB formed by N36 and C34-FITC. When ADS-J1 was added into the mixture, the upper band disappeared and was replaced by a lower band which was at the same position as C34-FITC alone (lane 4), confirming that ADS-J1 could disrupt the 6-HB formation between N36 and C34-FITC. Like N36, C60 alone did not display a band in the gel because it also carries net positive charge, rendering it unable to run into the native gel (lane 5). The N36/C34-FITC/C60 mixture exhibited a band at a higher position in lane 6 than the 6-HB band in lane 3 where the N36/C34-FITC mixture was loaded, indicating that C60 does not inhibit the 6-HB formation, but rather binds to 6-HB to form a new complex. Like N36, C60 alone did not display a band in line 1, 4 and 5 on the Coomassie Blue-stained gel because N36 and C60, having a large number of positive charges, could not run into to the native gel.

Using ELISA and C34 as the control 6-HB inhibitor, we further analyzed the potential inhibitory activity of C60 on 6-HB formation. As shown in Fig. 6B, C34 could compete with C34-biotin in binding to N36 and inhibited, in a dose-dependent manner, the 6-HB formed between N36 and C34-biotin, while C60 exhibited no significant inhibition at the concentration as high as 4 µM. In order to exclude the possibility that C60 may bind to a region other than the N36 sequence in gp41 NHR domain, we used N63, which covers the entire sequence of NHR, to repeat the above experiment. As shown in Fig. 6C, C60 could not inhibit formation of the 6-HB formed between N63 and C34-biotin, consistent with the result from the experiment using N36.

## Discussion

The HIV-1 gp41 6-HB core structure has been recognized as a critical structure in the viral fusion and entry process through the plasma membrane fusion or endocytosis pathways [17], [18]. Peptides derived from the gp41 CHR domain, such as SJ-2176 [42], C34 [19], [43] and T20 [44], can bind to the viral gp41 NHR domain to form heterogeneous 6-HB and block viral gp41 homologous 6-HB core formation in the target cell plasma membrane or endosomal membranes [45], [46]. However, it is unclear whether the HIV-1 gp41 6-HB core can serve as a target for developing HIV fusion inhibitors since it is believed that 6-HB is a dead-end structure in the HIV fusion process.

In this study, we conducted a yeast two-hybrid screen using the rsgp41 as the bait. We found that the human POB1 is able to bind to the HIV-1 gp41. Truncation analysis of gp41 and POB1 revealed that the binding sites of these two proteins were located at the C-terminal coiled-coil domain of POB1 (C60, aa 462–521) and the gp41 6-HB core formed by the NHR and CHR domain, respectively. Since the gp41 6-HB has been recognized as the dead-end structure formed in the HIV-1 fusion process, 6-HB-binding molecules are generally expected to be ineffective in inhibiting HIV-1 Env-mediated membrane fusion. Strikingly, however, the polypeptide C60 exhibited significant inhibition on HIV-1 Env-mediated cell fusion and infection by HIV-1 IIIB and Bal strains, with IC_50_ values at low µM level. Because C60 is derived from a human protein, it is not expected to induce antibody response against C60. Therefore, C60 can be used as a lead for development of safe and effective anti-HIV-1 therapeutics or microbicides for the treatment and prevention of HIV-1 infection.

**Figure 7:**
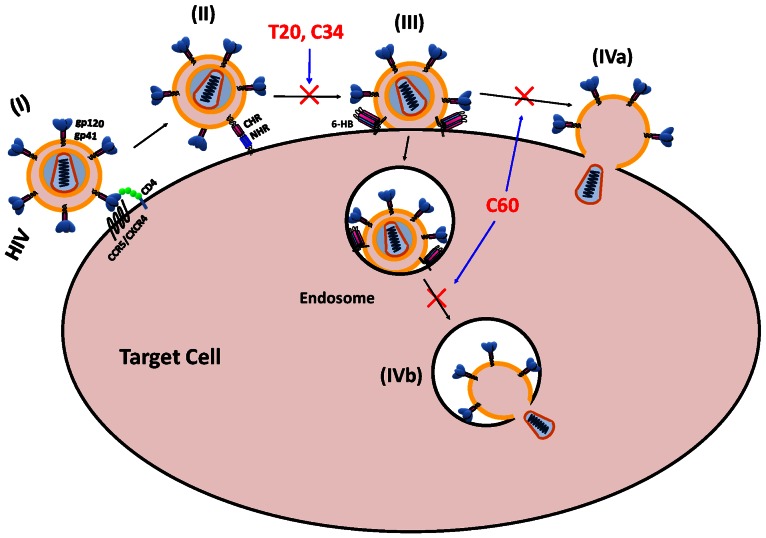
HIV-1 gp41 6-HB as a target for fusion inhibitor design. (**I**) The HIV-1 Env surface subunit gp120 binds with CD4 and co-receptor CCR5 or CXCR4 on the target cell. (**II**) The HIV-1 Env transmembrane subunit gp41 changes conformation to the pre-hairpin intermediate state with the exposed NHR-trimer, to which the CHR-peptides, such as C34 and T20, can bind, resulting in the inhibition of the 6-HB formation. (**III**) The HIV-1 gp41 refolds into the 6-HB conformation. (**IV**) The HIV-1 envelope can fuse with either the cytoplasmic membrane (IVa) through the plasma membrane fusion route or endosomal membrane (IVb) via the endocytosis pathway. The peptide C60 can bind with the 6-HB to stop the further process of fusion between viral envelope and plasma membrane (IVa) or endosomal membrane (IVb).

It has long been recognized that HIV-1 enters into the CD4+ T lymphocytes through the cytoplasmic membrane fusion in a pH-independent manor. However, this hypothesis has been challenged by researchers who have demonstrated that the entry of HIV-1 into the target cells is mainly through endocytosis [21]. It also raises questions about the actual role of the gp41 6-HB in the membrane fusion process since 6-HB is formed immediately after fusion pore formation in the endosomal membrane [20]. Consequently, the hypothesis that gp41 CHR-peptide-based HIV-1 fusion inhibitors inhibit HIV-1 fusion by binding to the gp41 NHR and subsequently blocking gp41 6-HB formation should be revisited [47]. Since C60 could bind the gp41 6-HB core and inhibit HIV-1 Env-mediated membrane fusion, it could also be used as a molecular probe to study the fusogenic mechanism of HIV-1 and determine the role of the gp41 6-HB core in the HIV-1 fusion process. We believe that unlike the CHR-peptide-based HIV fusion inhibitors such as C34 and T20, C60 inhibits fusion between the viral envelope and the cytoplasmic membrane or endosomal membrane by interacting with the gp41 6-HB (Fig. 7).

POB1 has been demonstrated to play an important role in EGF/insulin-induced endocytosis by coupling RalBP1 to Eps15 and Epsin, which bind to the AP-2 and clathrin complex [48]. Since clathrin is able to mediate endocytosis involving in HIV-1 entry [49], a rational question is whether C60 may interact with some cellular proteins, such as clathrin or dynamin, to indirectly affect the endocytosis of HIV-1. To answer this question, we pretreated target cells with C60 or MiTMAB, which is a inhibitor of clathrin-mediated endocytosis. After removal of the unbound inhibitors, we determined the susceptibility of these treated and untreated cells to the HIV-1 Bal infection. The results showed that MiTMAB-pretreated cells became less susceptible to HIV-1 infection, while C60-pretreated cells retain their susceptible to HIV-1. These results suggest that C60 may not regulate the endocytosis of HIV-1 through a cellular protein, but rather directly inhibits HIV-1 fusion with the target cell membranes by interacting with the viral proteins, such as gp41.

A number of the human restriction factors against HIV-1 replication, such as APOBEC3F, APOBEC3G, TRIM5alpha, Tetherin, and HERC5, have been identified [8]–[13]. These human proteins may be used as leads to develop anti-HIV-1 agents. However, none of these restriction factors targets the early stages of the HIV-1 life cycle, i.e., viral fusion and entry. Here we found that the C-terminal domain of human POB1 can bind to the gp41 6-HB core and inhibit HIV-1 Env-mediated membrane fusion at the late stage. It is, therefore, worthwhile investigating whether POB1 may also act as a host restriction factor to suppress HIV-1 fusion and entry through the endocytosis.

As shown in Fig. 3C, TZM-bl cells pre-treated with C60 are still susceptible to HIV-1 Bal infection, while the cells pretreated with MiTMAB (a inhibitor of clathrin-mediated endocytosis) became resistant to the viral infection, suggesting that unlike MiTMAB, C60 does not interact with a cellular protein (e.g., clathrin or dynamin) to interfere with the cellular protein-regulated endocytosis process. Rather, it may directly interact with the viral protein (e.g., gp41) to inhibit viral fusion with the target cell membranes. The entry of vesicular stomatitis virus (VSV) into the target cells was through the clathrin-based and dynamin-dependent endocytosis, and was sensitive to chlorpromazine (a inhibitor of clathrin-mediated endocytosis) and dynasore (an inhibitor of dynamin) [50], [51]. However, C60 exhibited no inhibition of VSV-G pseudoviruses infection, further suggesting that C60 may not interact with those proteins involving in endocytosis processes.

## References

[1] UNAIDS WHO (2009) AIDS epidemic update: November 2009. UNAIDS.

[2] Ammaranond P, Sanguansittianan S (2012) Mechanism of HIV antiretroviral drugs progress toward drug resistance. Fundam Clin Pharmacol 26: 146–161. 22118474 10.1111/j.1472-8206.2011.01009.x

[3] Wild C, Dubay JW, Greenwell T, Baird T Jr, Oas TG, et al (1994) Propensity for a leucine zipper-like domain of human immunodeficiency virus type 1 gp41 to form oligomers correlates with a role in virus-induced fusion rather than assembly of the glycoprotein complex. Proc Natl Acad Sci U S A 91: 12676–12680. 7809100 10.1073/pnas.91.26.12676 PMC45502

[4] Kilby JM, Hopkins S, Venetta TM, DiMassimo B, Cloud GA, et al (1998) Potent suppression of HIV-1 replication in humans by T-20, a peptide inhibitor of gp41-mediated virus entry. Nat Med 4: 1302–1307. 9809555 10.1038/3293

[5] Lalezari JP, Henry K, O'Hearn M, Montaner JS, Piliero PJ, et al (2003) Enfuvirtide, an HIV-1 fusion inhibitor, for drug-resistant HIV infection in North and South America. N Engl J Med 348: 2175–2185. 12637625 10.1056/NEJMoa035026

[6] Lazzarin A, Clotet B, Cooper D, Reynes J, Arasteh K, et al (2003) Efficacy of enfuvirtide in patients infected with drug-resistant HIV-1 in Europe and Australia. N Engl J Med 348: 2186–2195. 12773645 10.1056/NEJMoa035211

[7] Vincent N, Tardy J-C, Livrozet J-M, Lucht F, Frésard A, et al (2005) Depletion in Antibodies Targeted to the HR2 Region of HIV-1 Glycoprotein gp41 in Sera of HIV-1-Seropositive Patients Treated With T20. JAIDS Journal of Acquired Immune Deficiency Syndromes 38: 254–262. 15735441

[8] Wolf D, Goff SP (2008) Host restriction factors blocking retroviral replication. Annu Rev Genet 42: 143–163. 18624631 10.1146/annurev.genet.42.110807.091704 PMC3598625

[9] Mbisa JL, Bu W, Pathak VK (2010) APOBEC3F and APOBEC3G inhibit HIV-1 DNA integration by different mechanisms. J Virol 84: 5250–5259. 20219927 10.1128/JVI.02358-09 PMC2863843

[10] Stremlau M, Owens CM, Perron MJ, Kiessling M, Autissier P, et al (2004) The cytoplasmic body component TRIM5alpha restricts HIV-1 infection in Old World monkeys. Nature 427: 848–853. 14985764 10.1038/nature02343

[11] Neil SJ, Zang T, Bieniasz PD (2008) Tetherin inhibits retrovirus release and is antagonized by HIV-1 Vpu. Nature 451: 425–430. 18200009 10.1038/nature06553

[12] Van Damme N, Goff D, Katsura C, Jorgenson RL, Mitchell R, et al (2008) The interferon-induced protein BST-2 restricts HIV-1 release and is downregulated from the cell surface by the viral Vpu protein. Cell Host Microbe 3: 245–252. 18342597 10.1016/j.chom.2008.03.001 PMC2474773

[13] Woods MW, Kelly JN, Hattlmann CJ, Tong JG, Xu LS, et al (2011) Human HERC5 restricts an early stage of HIV-1 assembly by a mechanism correlating with the ISGylation of Gag. Retrovirology 8: 95. 22093708 10.1186/1742-4690-8-95 PMC3228677

[14] Sattentau QJ, Moore JP (1993) The Role of Cd4 in HIV Binding and Entry. Philos T Roy Soc B 342: 59–66. 10.1098/rstb.1993.0136 7904348

[15] Berger EA, Murphy PM, Farber JM (1999) Chemokine receptors as HIV-1 coreceptors: Roles in viral entry, tropism, and disease. Annu Rev Immunol 17: 657–700. 10358771 10.1146/annurev.immunol.17.1.657

[16] Chan DC, Kim PS (1998) HIV entry and its inhibition. Cell 93: 681–684. 9630213 10.1016/s0092-8674(00)81430-0

[17] Chan DC, Fass D, Berger JM, Kim PS (1997) Core structure of gp41 from the HIV envelope glycoprotein. Cell 89: 263–273. 9108481 10.1016/s0092-8674(00)80205-6

[18] Weissenhorn W, Dessen A, Harrison SC, Skehel JJ, Wiley DC (1997) Atomic structure of the ectodomain from HIV-1 gp41. Nature 387: 426–430. 9163431 10.1038/387426a0

[19] Lu M, Blacklow SC, Kim PS (1995) A Trimeric Structural Domain of the Hiv-1 Transmembrane Glycoprotein. Nat Struct Biol 2: 1075–1082. 8846219 10.1038/nsb1295-1075

[20] Markosyan RM, Cohen FS, Melikyan GB (2003) HIV-1 envelope proteins complete their folding into six-helix bundles immediately after fusion pore formation. Mol Biol Cell 14: 926–938. 12631714 10.1091/mbc.E02-09-0573 PMC151570

[21] Miyauchi K, Kim Y, Latinovic O, Morozov V, Melikyan GB (2009) HIV Enters Cells via Endocytosis and Dynamin-Dependent Fusion with Endosomes. Cell 137: 433–444. 19410541 10.1016/j.cell.2009.02.046 PMC2696170

[22] Chang LJ, Urlacher V, Iwakuma T, Cui Y, Zucali J (1999) Efficacy and safety analyses of a recombinant human immunodeficiency virus type 1 derived vector system. Gene Ther 6: 715–728. 10505094 10.1038/sj.gt.3300895

[23] Guo Y, Rumschlag-Booms E, Wang J, Xiao H, Yu J, et al (2009) Analysis of hemagglutinin-mediated entry tropism of H5N1 avian influenza. Virol J 6: 39. 19341465 10.1186/1743-422X-6-39 PMC2679739

[24] Zhu Y, Lu L, Xu L, Yang H, Jiang S, et al (2010) Identification of a gp41 core-binding molecule with homologous sequence of human TNNI3K-like protein as a novel human immunodeficiency virus type 1 entry inhibitor. J Virol 84: 9359–9368. 20592080 10.1128/JVI.00644-10 PMC2937606

[25] Chen X, Lu L, Qi Z, Lu H, Wang J, et al (2010) Novel recombinant engineered gp41 N-terminal heptad repeat trimers and their potential as anti-HIV-1 therapeutics or microbicides. J Biol Chem 285: 25506–25515. 20538590 10.1074/jbc.M110.101170 PMC2919114

[26] Liu ZQ, Chen YH (2004) Design and construction of a recombinant epitope-peptide gene as a universal epitope-vaccine strategy. J Immunol Methods 285: 93–97. 14871538 10.1016/j.jim.2003.10.018

[27] Zhu Y, Lu L, Chao L, Chen YH (2007) Important changes in biochemical properties and function of mutated LLP12 domain of HIV-1 gp41. Chem Biol Drug Des 70: 311–318. 17850282 10.1111/j.1747-0285.2007.00564.x PMC7188357

[28] Lu L, Zhu Y, Huang J, Chen X, Yang H, et al (2008) Surface exposure of the HIV-1 env cytoplasmic tail LLP2 domain during the membrane fusion process: interaction with gp41 fusion core. J Biol Chem 283: 16723–16731. 18408000 10.1074/jbc.M801083200 PMC2423246

[29] Jiang S, Lin K, Lu M (1998) A conformation-specific monoclonal antibody reacting with fusion-active gp41 from the human immunodeficiency virus type 1 envelope glycoprotein. J Virol 72: 10213–10217. 9811763 10.1128/jvi.72.12.10213-10217.1998 PMC110570

[30] Liu S, Zhao Q, Jiang S (2003) Determination of the HIV-1 gp41 fusogenic core conformation modeled by synthetic peptides: applicable for identification of HIV-1 fusion inhibitors. Peptides 24: 1303–1313. 14706544 10.1016/j.peptides.2003.07.013

[31] Jiang S, Lin K, Zhang L, Debnath AK (1999) A screening assay for antiviral compounds targeted to the HIV-1 gp41 core structure using a conformation-specific monoclonal antibody. J Virol Methods 80: 85–96. 10403680 10.1016/s0166-0934(99)00041-5

[32] Wang H, Qi Z, Guo A, Mao Q, Lu H, et al (2009) ADS-J1 inhibits human immunodeficiency virus type 1 entry by interacting with the gp41 pocket region and blocking fusion-active gp41 core formation. Antimicrob Agents Chemother 53: 4987–4998. 19786602 10.1128/AAC.00670-09 PMC2786362

[33] Qi Z, Shi W, Xue N, Pan C, Jing W, et al (2008) Rationally designed anti-HIV peptides containing multifunctional domains as molecule probes for studying the mechanisms of action of the first and second generation HIV fusion inhibitors. J Biol Chem 283: 30376–30384. 18662985 10.1074/jbc.M804672200 PMC2573079

[34] Jiang S, Lu H, Liu S, Zhao Q, He Y, et al (2004) N-substituted pyrrole derivatives as novel human immunodeficiency virus type 1 entry inhibitors that interfere with the gp41 six-helix bundle formation and block virus fusion. Antimicrob Agents Chemother 48: 4349–4359. 15504864 10.1128/AAC.48.11.4349-4359.2004 PMC525433

[35] Chou TC, Talalay P (1984) Quantitative analysis of dose-effect relationships: the combined effects of multiple drugs or enzyme inhibitors. Adv Enzyme Regul 22: 27–55. 6382953 10.1016/0065-2571(84)90007-4

[36] Neurath AR, Strick N, Jiang S, Li YY, Debnath AK (2002) Anti-HIV-1 activity of cellulose acetate phthalate: synergy with soluble CD4 and induction of "dead-end" gp41 six-helix bundles. BMC Infect Dis 2: 6. 11983022 10.1186/1471-2334-2-6 PMC113252

[37] Li L, He L, Tan S, Guo X, Lu H, et al (2010) 3-hydroxyphthalic anhydride-modified chicken ovalbumin exhibits potent and broad anti-HIV-1 activity: a potential microbicide for preventing sexual transmission of HIV-1. Antimicrob Agents Chemother 54: 1700–1711. 20194691 10.1128/AAC.01046-09 PMC2863607

[38] Ikeda M, Ishida O, Hinoi T, Kishida S, Kikuchi A (1998) Identification and characterization of a novel protein interacting with Ral-binding protein 1, a putative effector protein of Ral. Journal of Biological Chemistry 273: 814–821. 9422736 10.1074/jbc.273.2.814

[39] Huang JH, Lu L, Lu H, Chen X, Jiang S, et al (2007) Identification of the HIV-1 gp41 core-binding motif in the scaffolding domain of caveolin-1. J Biol Chem 282: 6143–6152. 17197700 10.1074/jbc.M607701200

[40] Root MJ, Kay MS, Kim PS (2001) Protein design of an HIV-1 entry inhibitor. Science 291: 884–888. 11229405 10.1126/science.1057453

[41] Moore JP, McKeating JA, Weiss RA, Sattentau QJ (1990) Dissociation of gp120 from HIV-1 virions induced by soluble CD4. Science 250: 1139–1142. 2251501 10.1126/science.2251501

[42] Jiang S, Lin K, Strick N, Neurath AR (1993) HIV-1 inhibition by a peptide. Nature 365: 113. 8371754 10.1038/365113a0

[43] Lu M, Kim PS (1997) A trimeric structural subdomain of the HIV-1 transmembrane glycoprotein. J Biomol Struct Dyn 15: 465–471. 9439994 10.1080/07391102.1997.10508958

[44] Wild CT, Shugars DC, Greenwell TK, McDanal CB, Matthews TJ (1994) Peptides corresponding to a predictive alpha-helical domain of human immunodeficiency virus type 1 gp41 are potent inhibitors of virus infection. Proc Natl Acad Sci U S A 91: 9770–9774. 7937889 10.1073/pnas.91.21.9770 PMC44898

[45] Chan DC, Kim PS (1998) HIV entry and its inhibition. Cell 93: 681–684. 9630213 10.1016/s0092-8674(00)81430-0

[46] Liu S, Wu S, Jiang S (2007) HIV entry inhibitors targeting gp41: from polypeptides to small-molecule compounds. Curr Pharm Des 13: 143–162. 17269924 10.2174/138161207779313722

[47] Miyauchi K, Kozlov MM, Melikyan GB (2009) Early steps of HIV-1 fusion define the sensitivity to inhibitory peptides that block 6-helix bundle formation. PLoS Pathog 5: e1000585. 19763181 10.1371/journal.ppat.1000585 PMC2736578

[48] Nakashima S, Morinaka K, Koyama S, Ikeda M, Kishida M, et al (1999) Small G protein Ral and its downstream molecules regulate endocytosis of EGF and insulin receptors. Embo Journal 18: 3629–3642. 10393179 10.1093/emboj/18.13.3629 PMC1171441

[49] Daecke J, Fackler OT, Dittmar MT, Krausslich HG (2005) Involvement of clathrin-mediated endocytosis in human immunodeficiency virus type 1 entry. J Virol 79: 1581–1594. 15650184 10.1128/JVI.79.3.1581-1594.2005 PMC544101

[50] Sun X, Yau VK, Briggs BJ, Whittaker GR (2005) Role of clathrin-mediated endocytosis during vesicular stomatitis virus entry into host cells. Virology 338: 53–60. 15936793 10.1016/j.virol.2005.05.006

[51] Johannsdottir HK, Mancini R, Kartenbeck J, Amato L, Helenius A (2009) Host cell factors and functions involved in vesicular stomatitis virus entry. J Virol 83: 440–453. 18971266 10.1128/JVI.01864-08 PMC2612308

